# Hemithyroidectomy *versus* total thyroidectomy for well differentiated T1–2 N0 thyroid cancer: systematic review and meta‐analysis

**DOI:** 10.1002/bjs5.50359

**Published:** 2020-10-06

**Authors:** P. M. Rodriguez Schaap, M. Botti, R. H. J. Otten, K. M. A. Dreijerink, E. J. M. Nieveen van Dijkum, H. J. Bonjer, A. F. Engelsman, C. Dickhoff

**Affiliations:** ^1^ Department of Surgery Amsterdam the Netherlands; ^2^ Department of Endocrinology Amsterdam the Netherlands; ^3^ Medical Library, Amsterdam University Medical Centre, location VUmc, Cancer Centre Amsterdam Amsterdam the Netherlands; ^4^ Department of Surgery, Amsterdam University Medical Centre location AMC Amsterdam the Netherlands; ^5^ Department of General Surgery University of Pavia, IRCSS Fondazione Policlinico San Matteo Pavia Italy

## Abstract

**Background:**

Evidence for limiting the extent of surgery in patients with low‐risk thyroid cancer is lacking.

**Methods:**

A systematic search was performed according to the PRISMA and MOOSE guidelines to assess the effect of total thyroidectomy (TT) with or without radioactive iodine (RAI) treatment *versus* hemithyroidectomy (HT) on recurrence and overall mortality in patients with differentiated (papillary or follicular) T1–2 N0 thyroid cancer. PubMed, Embase and Cochrane databases were searched, and two authors independently assessed the articles.

**Results:**

A total of ten eligible articles were identified. All were observational cohort series, representing a total of 23 134 patients, of which 17 699 were available for meta‐analysis. Six studies included patients who had TT followed by RAI treatment. The pooled recurrence rate after TT ± RAI and HT was 2·3 and 2·8 per cent respectively (odds ratio (OR) 1·12, 95 per cent c.i. 0·82 to 1·53; *P* = 0·48). The pooled 20‐year overall survival rate after TT ± RAI was 96·8 per cent, compared with 97·4 per cent for HT (OR 1·30, 0·71 to 2·37; *P* = 0·40). Overall, higher complication rates were found in the TT ± RAI group.

**Conclusion:**

Recurrence rates after HT for treatment of well differentiated T1–2 N0 thyroid cancer were similar to those after TT ± RAI, with a lower incidence of treatment‐related complications.

## Graphical abstract

This systematic review and meta‐analysis showed similar recurrence rates after hemithyroidectomy for selected patients with well differentiated T1–2 N0 thyroid cancer to those in patients who had a total thyroidectomy with or without radioactive iodine treatment, with lower reported rates of treatment‐related complications.

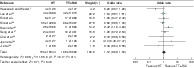

Supports conservative surgery

## Abstract (es)

**Antecedentes:**

No hay evidencia para limitar la extensión de la cirugía en pacientes con cáncer de tiroides de bajo riesgo.

**Métodos:**

Se realizó una búsqueda sistemática siguiendo las recomendaciones PRISMA (*Preferred Reporting Items for Systematic Reviews and Meta‐analysis*) y MOOSE (*Meta‐analysis of Observational Studies in Epidemiology*) para evaluar el efecto de la tiroidectomía total (TT) +/− yodo radioactivo (*radioactive iodine treatment*, RAI) *versus* hemitiroidectomía (HT) en la recidiva y en la mortalidad global en el cáncer de tiroides diferenciado (papilar/folicular) T1‐T2N0. Se realizaron búsquedas en las bases de datos PubMed, Embase y Cochrane, y dos autores evaluaron los artículos de forma independiente.

**Resultados:**

Se identificaron un total de 10 artículos de interés. Todos ellos eran estudios de cohortes observacionales, con un total de 23.134 pacientes, de los cuales 17.699 se incluyeron en el metaanálisis. En seis estudios, los pacientes fueron tratados mediante TT seguida de RAI. Las tasas agrupadas de recidiva tras TT +/− RAI y HT fueron 2,3% and 2,8%, respectivamente (razón de oportunidades, *odds ratio*, OR = 1,12, i.c. del 95% 0,82‐1,54, *P* = 0.48). La supervivencia global a 20 años de TT +/− RAI fue del 96,8% en comparación con el 97,4% para la HT (OR = 1,30, i.c. del 95% 0,71‐2,37, *P* = 0,40). Globalmente, se observaron más complicaciones en el grupo de TT +/− RAI.

**Conclusión:**

Esta revisión sistemática con metaanálisis demuestra tasas de recidiva similares tras una HT para el tratamiento del cáncer de tiroides T1‐2N0 bien diferenciado en comparación con TT +/− RAI, con una menor incidencia de complicaciones relacionadas con el tratamiento.

## Introduction

The incidence of thyroid cancer has been increasing over the past three decades, affecting both sexes and all ages^1^, ^2^. The standard of care for well differentiated thyroid cancer is total thyroidectomy (TT) and adjuvant radioactive iodine (RAI) treatment, resulting in reported 10‐year overall survival rates of 96–98 per cent^3^. Apart from a lifelong need for thyroid hormone supplementation, complications such as recurrent laryngeal nerve damage, bleeding and hypoparathyroidism contribute to treatment‐related morbidity and decreased quality of life^4^, ^5^.

Large non‐comparative national database cohort studies^6^, ^7^, ^8^, ^9^, however, show neither survival benefit nor difference in recurrence rates between TT followed by RAI and hemithyroidectomy (HT) for well differentiated, low‐risk thyroid cancer. These low recurrence rates and good long‐term survival explain an increased interest in de‐escalation of treatment for these patients^10^. Recently, the American Thyroid Association^11^ recommended consideration of HT for selected patients with low‐risk, well differentiated thyroid cancer.

**Fig. 1:**
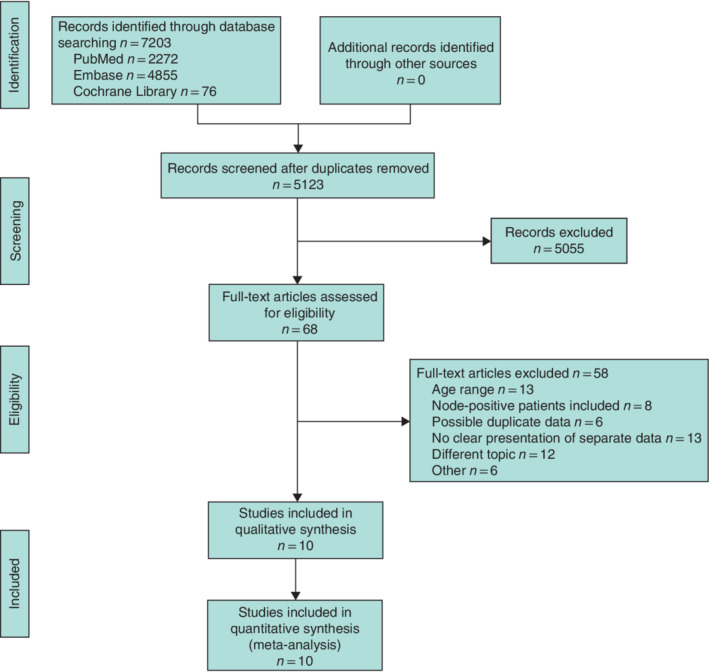
PRISMA diagram for the review

In selected patients, less extensive surgery for subcentimetre papillary thyroid microcarcinoma (PTMC) is supported by data from large databases^12^, ^13^, but it is unclear whether HT will result in similar treatment outcomes in patients with low‐risk differentiated thyroid cancers larger than 1 cm. This systematic review and meta‐analysis investigated HT as the definitive treatment for patients with low‐risk, well differentiated (papillary or follicular), node‐negative thyroid cancer of 4 cm or less (T1–2 N0), with a particular focus on recurrence and overall survival.

## Methods

The study was registered in the International Prospective Register of Systematic Reviews (PROSPERO) (registration number CRD42018115971). A literature search was performed based on the PRISMA^14^ and MOOSE^15^ guidelines. To identify all relevant publications on HT for differentiated T1–2 N0 thyroid cancer, systematic searches in the bibliographic databases PubMed, EMBASE.com and the Cochrane Library (via Wiley) (*Tables S1–S3*, supporting information) were performed from inception to 11 November 2019. Search terms included controlled terms (Medical Subject Headings (MeSH) in PubMed and Emtree in Embase), as well as free text terms in the Cochrane Library. Searches focused on differentiated thyroid cancer (papillary and follicular), tumour size 0–4 cm, and HT compared with TT ± RAI.

After removal of duplicates, articles were initially screened by title and abstract to exclude irrelevant reports. Of the remaining articles, full texts were screened for eligibility by two authors. Reference lists of relevant articles were searched. Any discrepancy regarding article selection was resolved by consensus.

### Inclusion and exclusion criteria

Any English‐language original reports comparing HT with TT ± RAI for the treatment of 0–4‐cm, well differentiated, low‐risk thyroid cancer were included. Low risk was defined as node‐negative (N0), T1 and T2 cancers^16^. Studies describing recurrent disease only, those including patients under 18 years of age, letters, reviews, editorials, case series with fewer than ten patients, and conference abstracts were excluded.

### Data extraction

The following characteristics were extracted from the included studies: patient demographics (number of patients, age and sex), pathology, treatment characteristics, tumour size and definition of recurrence. The primary endpoint was recurrence rate and time to recurrence. Secondary endpoints were overall survival and perioperative morbidity, including laryngeal nerve damage, hypoparathyroidism and hypocalcaemia.

### Statistical analysis

For the meta‐analysis, outcome data for recurrence and overall survival were pooled using Review Manager version 5.3 (The Nordic Cochrane Centre, The Cochrane Collaboration, Copenhagen, Denmark) and presented as forest plots. Heterogeneity was assessed by calculating the *I* ^2^ index. For low heterogeneity (*I* ^2^ below 25 per cent), a fixed‐effect model was used for meta‐analysis. For intermediate heterogeneity (*I* ^2^ ranging from 25 to 75 per cent), a random‐effects model was used for meta‐analysis. *I* ^2^ above 75 per cent was considered substantial, and no meta‐analysis was performed. Where available, data from matched‐pair analysis of low‐risk patients were used for the meta‐analysis.

**Fig. 2:**
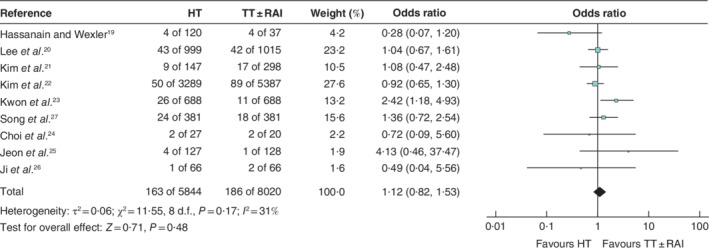
Forest plot comparing recurrence after hemithyroidectomy or total thyroidectomy with or without radioactive iodine treatment A Mantel–Haenszel random‐effects model was used for meta‐analysis. Odds ratios are shown with 95 per cent confidence intervals. HT, hemithyroidectomy; TT, total thyroidectomy; RAI, radioactive iodine.

**Fig. 3:**
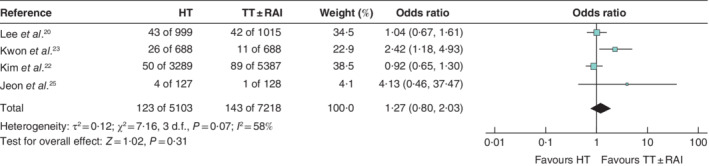
Forest plot comparing recurrence after hemithyroidectomy or total thyroidectomy with or without radioactive iodine treatment for the subgroup analysis of papillary thyroid microcarcinoma (0–1 cm) A Mantel–Haenszel random‐effects model was used for meta‐analysis. Odds ratios are shown with 95 per cent confidence intervals. HT, hemithyroidectomy; TT, total thyroidectomy; RAI, radioactive iodine.

**Fig. 4:**
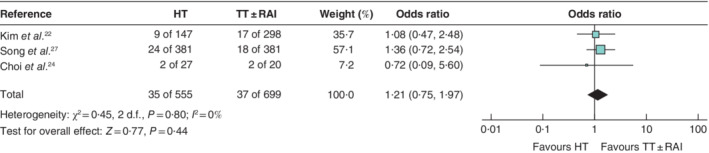
Forest plot comparing recurrence after hemithyroidectomy or total thyroidectomy with or without radioactive iodine treatment for the subgroup analysis of papillary thyroid carcinoma (1–4 cm) A Mantel–Haenszel fixed‐effect model was used for meta‐analysis. Odds ratios are shown with 95 per cent confidence intervals. HT, hemithyroidectomy; TT, total thyroidectomy; RAI, radioactive iodine.

### Quality assessment and risk of bias

The Risk Of Bias In Non‐randomized Studies of Interventions (ROBINS‐I) tool^17^ was used to score the overall risk of bias. Funnel plots were made to assess publication bias.

## Results

The initial search resulted in a total of 7203 records. After removal of 2080 duplicates and screening of all titles and abstracts, a total of 68 papers were assessed in full text for inclusion, after which 58 were excluded, resulting in a total of ten studies^18^, ^19^, ^20^, ^21^, ^22^, ^23^, ^24^, ^25^, ^26^, ^27^ for analysis (*Fig*. *1*).

All ten articles described observational cohorts, representing a total of 23 134 patients, predominantly women: 19 069 women (82·4 per cent) and 4065 men (17·6 per cent). Some 17 699 patients with low‐risk, well differentiated thyroid cancer were included in the meta‐analysis. No prospective randomized studies were available (*Table* *1*). The reported duration of follow‐up ranged from 57·3 to 141·6 months. To assess outcome, five studies^20^, ^21^, ^23^, ^26^, ^27^ performed a matched‐pair analysis based on patient characteristics.

**Table 1:** Characteristics of included studies

Reference	Inclusion period	Country	Duration of follow‐up (years)*	No. of patients	AMES criteria (low *versus* high risk)	No. in HT group	No. in TT ± RAI group	Matched‐pair analysis
Cross *et al*.^18^	1940–1998	USA	15·3	962	746 low risk 207 high risk	237 (179 for analysis)	716(567 for analysis)	n.a.
Hassanain and Wexler^19^	1982–2002	Canada	10 (4–25)†	180	161 low risk 17 high risk	126	54	n.a.
Lee *et al*.^20^	1986–2006	Korea	11·8 (5–26)†	2014	n.a.	999	1015	506 HT506 TT
Kim *et al*.^21^	2004–2008	Korea	7·0	1150	n.a.	147	1003	147 HT298 TT
Kim *et al*.^22^	1997–2015	South Korea	5·4 (0·5–19·2)†	8676	n.a.	3289	5387	n.a.
Kwon *et al*.^23^	1998–2007	Korea	8·5	2031	n.a.	755	1276	688 HT688 TT
Choi *et al*.^24^	1978–2011	Korea	4·8(4·8)	5266	47 low risk 5219 high risk	974 (27 for analysis)	4292 (20 for analysis)	n.a.
Jeon *et al*.^25^	1999–2012	Korea	7·9 (2–17·4)†	255	255 low risk	127	128	n.a.
Ji *et al*.^26^	2001–2014	Korea	5·5	255	n.a.	82	173	66 HT66 TT
Song *et al*.^27^	1998–2007	Korea	9·8	2345	n.a.	383	1962	381 HT381 TT

All studies had a retrospective design.

* Values are mean(s.d.) unless indicated otherwise;

† values are median (range). AMES, Age, Metastases, Extent and Size (risk classification); HT, hemithyroidectomy; TT, total thyroidectomy; RAI, radioactive iodine; n.a., not available.

Patient characteristics are summarized in *Table* *2*. The median age ranged from 43 to 49 years. From seven studies^19^, ^20^, ^22^, ^23^, ^24^, ^25^, ^26^ that explicitly reported histopathological characteristics, 13 412 patients with papillary thyroid cancer (PTC) and 40 with follicular thyroid cancer (FTC) were included. One study^18^ reported on patients with PTC and FTC, but did not provide separate data on histopathological characteristics for those in the low‐risk treatment group, and two studies^21^, ^27^ failed to report histopathological characteristics. Tumour size ranged from less than 0·5 to 1·93 cm. RAI treatment following TT surgery was reported in six studies^21^, ^22^, ^23^, ^24^, ^26^, ^27^ and varied from 71·7 to 93·9 per cent of the cohorts.

**Table 2:** Characteristics of patients in included studies

Reference	No. of patients in meta‐analysis	Sex ratio (F : M)	Age (years)*	Pathology	Tumour size*	% of patients in TT group treated with RAI	Recurrence (HT group *versus* TT ± RAI group) (%)	20‐year overall survival in low‐risk group (HT *versus* TT ± RAI) (%)
Cross *et al*.^18^	746	260 : 702	43†	PTC, FTC	n.a.	n.a.	n.a.	98·5 *versus* 97·0
Hassanain and Wexler^19^	180	131 : 49	n.a.	PTC (150), FTC (24), other (6)	n.a.	n.a.	3 *versus* 11 (low‐risk group)	n.a. 5 deaths (2·8%), 4 within first year of diagnosis, all high risk; no analysis done
Lee *et al*.^20^	2014	1827 : 187	HT: 43·9† TT: 47·7†	PTMC	< 0·5 cm in 894 and > 0·5 cm in 1120 → 5·7 mm in HT group *versus* 6·9 mm in TT group	n.a.	1·9, 4·3, 6·5 *versus* 2·3, 4·1, 5·9(LRR at 5,10 and 20 years respectively)	97·1 *versus* 96·6
Kim *et al*.^21^	1150	1073 : 77	44(11)	n.a.	1·6(0·6) cm	74	6·1 *versus* 5·7	n.a.
Kim *et al*.^22^	8676	7057 : 1619	47·2(10·5)	PTMC	0·6(0·2) cm	71·7	1·5 *versus* 1·7	n.a.
Kwon *et al*.^23^	2031	1790 : 241	After matching: 47 (41–54)†	PTMC	After matching: 0·6 cm	87	3·8 *versus* 1·6	n.a.
Choi *et al*.^24^	47	4462 : 804	45·41(12·10)	PTC (31), minimally invasive FTC (16)	HT: 1·60(0·56) cm TT: 1·93(0·81) cm	92·7	7·4 *versus* 10 (low‐risk group, *n* = 47)	n.a.
Jeon *et al*.^25^	255	226 : 29	49 (25–76)†	PTMC	6·49 (1–10) mm†	Postop. RAI was exclusion criterion (0)	3·15 *versus* 0·78	100 *versus* 100
Ji *et al*.^26^	255	201 : 54	After matching: HT: 44·6(11·2) TT: 45·2(11·0)	PTC	After matching: HT: 7·2(3·1) mm TT: 7·3(3·1) mm	92·4	97·9 *versus* 96·5	n.a.
Song *et al*.^27^	2345	2042 : 303	After matching: HT: 44·0 (37·3–52·5)† TT: 46·9 (38·0–53·9)†	n.a.	HT: 1·4(0·6) cm TT: 1·8(0·7) cm	93·9	6·3 *versus* 4·7	n.a.

* Values are mean(s.d.) unless indicated otherwise;

† values are median (range). TT, total thyroidectomy; RAI, radioactive iodine; HT, hemithyroidectomy; PTC, papillary thyroid carcinoma; FTC, follicular thyroid carcinoma; n.a., not available; PTMC, papillary thyroid microcarcinoma; LRR, locoregional recurrence.

### Recurrence

Nine studies^19^, ^20^, ^21^, ^22^, ^23^, ^24^, ^25^, ^26^, ^27^, including 13 864 patients, reported recurrence rates. The pooled recurrence rates after TT ± RAI and HT were 2·3 and 2·8 per cent respectively (odds ratio (OR) 1·12, 95 per cent c.i. 0·82 to 1·53; *P* = 0·48) (*Fig*. *2*). Median time to recurrence was reported in four studies^19^, ^23^, ^24^, ^25^ and ranged from 3·58 to 5·62 years. The majority of studies defined recurrence as proven by cytology and/or pathology (*Table S4*, supporting information). A total of four studies^20^, ^22^, ^23^, ^25^, involving 12 321 patients, reported on recurrence in patients with PTMC. The pooled recurrence rate after TT ± RAI was 2·0 per cent, compared with a pooled recurrence rate of 2·4 per cent for HT (OR 1·27, 0·80 to 2·03; *P* = 0·31) (*Fig*. *3*). In addition, three studies^21^, ^24^, ^27^, involving 1254 patients, reported recurrence rates after surgery for well differentiated thyroid cancers of 1–4 cm. The pooled recurrence rate after TT ± RAI was 5·3 per cent *versus* 6·3 per cent for HT (OR 1·21, 0·75 to 1·97; *P* = 0·44) (*Fig*. *4*).

### Overall survival

Only two studies^18^, ^20^, including 1758 patients, reported overall survival. The pooled 20‐year overall survival rate after TT ± RAI was 96·8 per cent, compared with 97·4 per cent for HT (OR 1·30, 95 per cent c.i. 0·71 to 2·37; *P* = 0·40) (*Fig. S1*, supporting information).

### Complications

Complications were reported in four studies^21^, ^23^, ^25^, ^26^ (*Table* *3*). Overall, higher complication rates were found in the TT ± RAI group. Bleeding was reported in two studies^23^, ^26^. In the HT group, bleeding rates were 0 per cent^26^ and 0·7 per cent^23^, compared with rates of 0 per cent^26^ (*P* = 1·0) and 1·0 per cent^23^ (*P* = 0·6) in the TT ± RAI group. Regarding laryngeal nerve injury, TT ± RAI had a higher rate than HT, with an incidence from 0 per cent^21^, ^26^ to 1·6 per cent^25^ for HT and from 0·6 per cent^23^ to 9·4 per cent^25^ for TT ± RAI. Permanent hypoparathyroidism was reported in three studies^21^, ^23^, ^26^. Although no patients with hypoparathyroidism were reported following HT, in the TT ± RAI groups the rate ranged from 1·7 per cent^23^ (*P* < 0·001) to 7·7 per cent^21^. Finally, one study^26^ reported on seroma, with 19 (29 per cent) and 25 (38 per cent) patients in the HT and TT ± RAI groups respectively (*P* = 0·268).

**Table 3:** Complications reported by studies included in the analysis

	Kim *et al*.^21^		Kwon *et al*.^23^, *			Jeon *et al*.^25^		Ji *et al*.^26^, *		
	HT	TT ± RAI	HT	TT ± RAI	*P* †	HT	TT	HT	TT ± RAI	*P* ‡
**Bleeding**	–	–	5 (0·7)	7 (1·0)	0·6	–	–	0 (0)	0 (0)	1·0
**RLN damage**										
Permanent	0 (0)	44 (4·4)	2 (0·3)	4 (0·6)	< 0·7	2 (1·6)	12 (9·4)	0 (0)	0 (0)	1·0
Transient	–	–	–	n	–	–	–	1 (2)	2 (3)	1·0
**Hypoparathyroidism**										
Permanent	0 (0)	77 (7·7)	0 (0)	12 (1·7)	< 0·001	–	–	0 (0)	2 (3	0·496
Transient	–	–	0 (0)	103 (15·0)	–	0 (0)	11 (8·6)	5 (8)	32 (48)	< 0·001
**Seroma**	–	–	–	–	–	–	–	19 (29)	25 (38)	0·268

Values in parentheses are percentages.

* Values from matched‐pair analysis with statistical comparison. HT, hemithyroidectomy; TT, total thyroidectomy; RAI, radioactive iodine; RLN, recurrent laryngeal nerve.

† χ^2^ test.

‡ Continuous variables compared with Student's *t* test, and categorical variables with Pearson's χ^2^ or Fisher's exact test.

### Quality assessment and risk of bias

Quality of included studies according to the ROBINS‐I tool is presented in *Table S5* (supporting information). The overall quality of the included studies was considered moderate to critical, based mainly on the observational nature of included studies. Funnel plots showed an even distribution of studies over possible outcomes, suggesting that publication bias was minimal (*Figs S2–S5*, supporting information).

## Discussion

This systematic review with meta‐analysis has shown that, for patients with low‐risk, well differentiated thyroid cancer, low recurrence rates and high survival can be achieved with both HT and TT ± RAI treatment. Reported recurrence and 20‐year survival rates were similar. These data correspond to survival rates from large national databases^12^, ^13^. The updated American Thyroid Association guideline^11^ recommending HT for selected patients with 1–4‐cm well differentiated PTC is supported by the results of the present meta‐analysis.

As the present results indicated that HT was a non‐inferior alternative to TT ± RAI for well differentiated T1–2 N0 thyroid cancer, possible complications of both treatments, such as recurrent nerve damage, hypoparathyroidism and hypothyroidism, need to be taken into account. Hypoparathyroidism, with rates ranging from 1·7 to 7·7 per cent, was seen exclusively in the TT ± RAI group. These rates are comparable with those reported for temporary and permanent postoperative hypoparathyroidism after TT, which have been described with a prevalence of 1·6–53·6 and 0·2–9·3 per cent respectively^28^, ^29^. Hypoparathyroidism after thyroidectomy has been shown to correlate with a decrease in overall survival, even in patients who had surgery for benign thyroid disease^30^. In addition, with HT a considerable proportion of patients could be prevented from having to take lifelong oral thyroid hormone substitution. Although after TT there is a 100 per cent risk of hypothyroidism, and supplementation is always needed, a small number of patients do need thyroxine supplements after HT^31^.

The follow‐up strategy is also important. Clearly, active follow‐up is warranted after HT so that, if recurrence or metastasis is identified, completion thyroidectomy can performed followed by RAI treatment if necessary. There are no guidelines outlining the duration and method of such follow‐up.

There are limitations when interpreting the results of this meta‐analysis. The absence of any randomized comparison is important, and the selection biases inherent in cohort studies are acknowledged. Importantly, it has to be noted that a large proportion of included patients in both HT and TT ± RAI groups had PTMC, limiting the evidence on treatment with HT for tumours sized 1–4 cm. Rates of recurrence and overall survival were, nevertheless, comparable for HT and TT ± RAI in the patients with 1–4‐cm tumours. Two of the included studies^18^, ^24^ involved patients from a considerable time ago, which may have resulted in an underestimation of the clinical N and M status as modern ultrasound and CT scans were not available at that time. Despite expecting that this might negatively affect the oncological outcome of patients who had HT, there was still no difference in oncological outcome in the pooled results of the meta‐analysis. Some of the studies included both patients with PTC and those with FTC^18^, ^19^, ^24^, and the prognosis for FTC depends mainly on the degree of invasiveness rather than primary tumour size. Unfortunately, data from studies reporting on both PTC and FTC were not presented separately. Additional limitations include the different definitions of both recurrence and complications used in the studies, and only two studies reported survival data.

## Disclosure

The authors declare no conflict of interest.

## Supporting information


**Appendix S1**: Supporting information
