# Supplementary material for: Hemithyroidectomy versus total thyroidectomy for well differentiated T1–2 N0 thyroid cancer: systematic review and meta‐analysis
Source: BJS Open. 2020 Oct 6;4(6):987–94. doi: 10.1002/bjs5.50359 (PMC7709359; doi:10.1002/bjs5.50359)

**BJS5_50359**

**Hemithyroidectomy *versus* total thyroidectomy for well differentiated T1–2 N0 thyroid cancer: systematic review and meta-analysis**

**P. M. Rodriguez Schaap, M. Botti, R. H. J. Otten, K. M. A. Dreijerink, E. J. M. Nieveen van Dijkum, H. J. Bonjer, A. F. Engelsman and C. Dickhoff**

**Table S1** Search strategy in PubMed (11 November 2019)

| **#** | **Query** | **Results** |
| --- | --- | --- |
| **#8** | #3 OR #5 OR #7 | **2272** |
| **#7** | #1 AND #6 | **686** |
| **#6** | unilateral thyroidectom*[tiab] OR subtotal thyroidectom*[tiab] OR partial thyroidectom*[tiab] | **1795** |
| **#5** | #1 AND #4 | **1274** |
| **#4** | lobectom*[tw] | **19207** |
| **#3** | #1 AND #2 | **556** |
| **#2** | hemithyroidectom*[tw] OR hemi-thyroidectom*[tw] | **1028** |
| **#1** | "Thyroid Neoplasms"[Mesh] OR thyroid neoplas*[tiab] OR thyroid cancer*[tiab] OR thyroid carcinoma*[tiab] OR thyroid tumor*[tiab] OR thyroid tumour*[tiab] | **61336** |

**Table S2** Search strategy in Embase.com (11 November 2019)

| **#** | **Query** | **Results** |
| --- | --- | --- |
| **#8** | #3 OR #5 OR #7 | **4855** |
| **#7** | #1 AND #6 | **2641** |
| **#6** | 'subtotal thyroidectomy'/exp OR 'unilateral thyroidectom*':ti,ab,kw OR 'subtotal thyroidectom*':ti,ab,kw OR 'partial thyroidectom*':ti,ab,kw | **4749** |
| **#5** | #1 AND #4 | **2295** |
| **#4** | 'lobectomy'/exp OR lobectom*:ti,ab,kw | **31606** |
| **#3** | #1 AND #2 | **969** |
| **#2** | 'hemithyroidectomy'/exp OR hemithyroidectom*:ti,ab,kw OR 'hemi thyroidectom*':ti,ab,kw | **1632** |
| **#1** | 'thyroid tumor'/exp OR 'thyroid neoplas*':ti,ab,kw OR 'thyroid cancer*':ti,ab,kw OR 'thyroid carcinoma*':ti,ab,kw OR 'thyroid tumor*':ti,ab,kw OR 'thyroid tumour*':ti,ab,kw | **89828** |

**Table S3** Search strategy in the Cochrane Library via Wiley (11 November 2019)

| **#** | **Query** | **Results** |
| --- | --- | --- |
| **#8** | #3 OR #5 OR #7 | **76** |
| **#7** | #1 AND #6 | **35** |
| **#6** | (unilateral NEXT thyroidectom*):ti,ab,kw OR (subtotal NEXT thyroidectom*):ti,ab,kw OR (partial thyroidectom*):ti,ab,kw | **140** |
| **#5** | #1 AND #4 | **33** |
| **#4** | lobectom*:ti,ab,kw | **1378** |
| **#3** | #1 AND #2 | **18** |
| **#2** | hemithyroidectom*:ti,ab,kw OR (hemi NEXT thyroidectom*):ti,ab,kw | **70** |
| **#1** | (thyroid NEXT neoplas*):ti,ab,kw OR (thyroid NEXT cancer*):ti,ab,kw OR (thyroid NEXT carcinoma*):ti,ab,kw OR (thyroid NEXT tumor*):ti,ab,kw OR (thyroid NEXT tumour*):ti,ab,kw | **1418** |

**Table S4** Follow-up protocols and definitions of recurrence

|  |  | Follow -up protocol |  | Recurrence defined by: |  |
| --- | --- | --- | --- | --- | --- |
| Author |  | Tg levels | Imaging | Tg levels only | Cytology/Pathology |
| **Cross et al.**^18^ |  | N/A | N/A | N/A | N/A |
| **Hassanain et al.**^19^ |  | N/A | N/A | N/A | N/A |
| **Lee et al.**^20^ |  | + | + | - | + |
| **Kim MJ et al.**^21^ |  | N/A | N/A | - | + |
| **Kim SK et al.**^22^ |  | + | + | - | + |
| **Kwon et al.**^23^ |  | - | + | - | + |
| **Choi et al.**^24^ |  | N/A | N/A | - | + |
| **Jeon et al.**^25^ |  | N/A | N/A | - | + |
| **Ji et al.**^26^ |  | - | + | - | + |
| **Song et al.**^27^ |  | + | + | - | + |

N/A: not available, Tg: Thyroglobulin

**Table S5** Risk of bias in included studies scored with the ROBINS-I tool

| **Author** | **Year of Publication** | **Bias due to confounding** | **Bias in selection of participants into the study** | **Bias in classification of interventions.** | **Bias due to deviations from intended interventions** | **Bias due to missing data** | **Bias in measurement of outcome** | **Bias in election of reported result** | **Overall risk of bias** |
| --- | --- | --- | --- | --- | --- | --- | --- | --- | --- |
| **Cross et al.**^18^ | 2006 | +/- | - | - | - | +/- | - | - | - |
| **Hassanain et al.**^19^ | 2010 | +/- | +/- | +/- | + | + | - | +/- | - |
| **Lee et al.**^20^ | 2013 | + | +/- | +/- | - | +/- | +/- | + | - |
| **Kim MJ et al.**^21^ | 2017 | + | + | +/- | +/- | +/- | +/- | +/- | +/- |
| **Kim SK et al.**^22^ | 2017 | +/- | +/- | + | +/- | +/- | +/- | +/- | +/- |
| **Kwon et al.**^23^ | 2017 | + | +/- | + | + | + | +/- | + | +/- |
| **Choi et al.**^24^ | 2019 | +/- | + | +/- | - | + | +/- | +/- | - |
| **Jeon et al.**^25^ | 2019 | +/- | - | +/- | +/- | + | +/- | +/- | - |
| **Ji et al.**^26^ | 2019 | + | +/- | +/- | +/- | - | +/- | + | - |
| **Song et al.**^27^ | 2019 | + | + | +/- | +/- | +/- | +/- | + | +/- |

+: low (green); +/-: moderate (yellow); -: critical (red)

**Fig. S1** Pooled data for overall survival after hemithyroidectomy or total thyroidectomy with or without radioactive iodine treatment


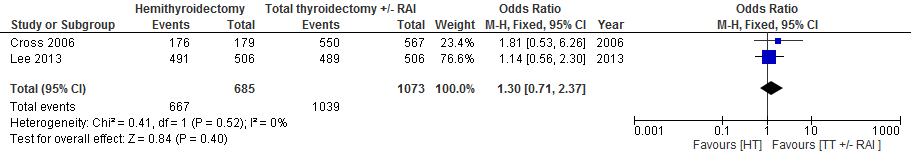


**Fig. S2** Funnel plot assessing publication bias for the meta-analysis of recurrence after hemithyroidectomy or total thyroidectomy with or without radioactive iodine treatment


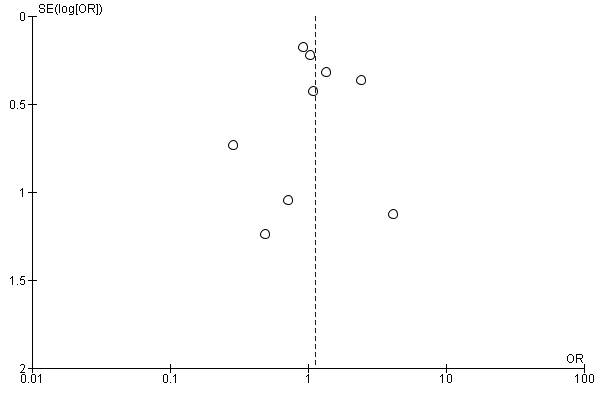


**Fig. S3** Funnel plot assessing publication bias for the meta-analysis of recurrence after hemithyroidectomy or total thyroidectomy with or without radioactive iodine treatment for the subgroup analysis of papillary thyroid microcarcinoma (0–1 cm)


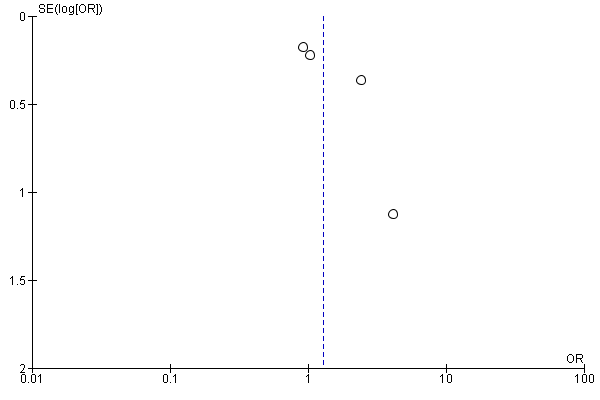


**Fig. S4** Funnel plot assessing publication bias for the meta-analysis of recurrence after hemithyroidectomy or total thyroidectomy with or without radioactive iodine treatment for the subgroup analysis of papillary thyroid carcinoma (1–4 cm)


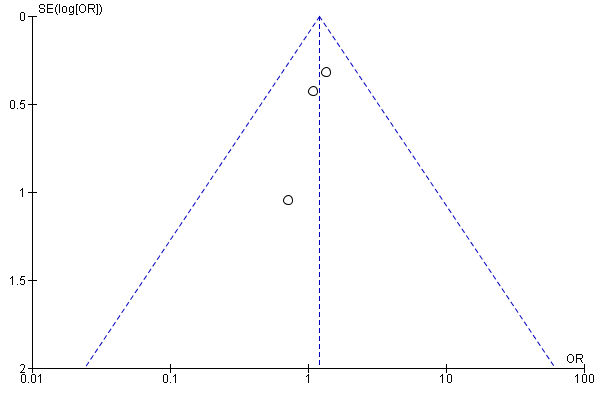


**Fig. S5** Funnel plot assessing publication bias for the meta-analysis of overall survival after hemithyroidectomy or total thyroidectomy with or without radioactive iodine treatment


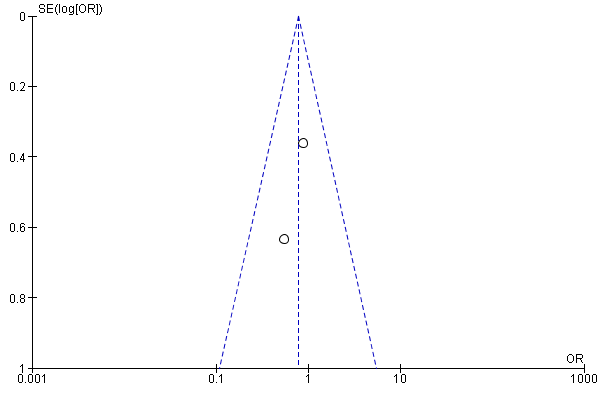

Supplement: Supplementary file 1 — Appendix S1: Supporting information [file BJS5-4-987-s001.docx]
